# Rapid Nitriding of Titanium Alloy with Fine Grains at Room Temperature

**DOI:** 10.1002/adma.202008298

**Published:** 2021-05-03

**Authors:** Keisuke Fujita, Masataka Ijiri, Yoichi Inoue, Shoichi Kikuchi

**Affiliations:** ^1^ Department of Engineering Graduate School of Integrated Science and Technology Shizuoka University 3‐5‐1 Johoku, Naka‐ku, Hamamatsu‐shi Shizuoka 4328561 Japan; ^2^ Department of Advanced Machinery Engineering School of Engineering Tokyo Denki University 5 Senju‐Asahi‐cho, Adachi‐ku Tokyo 1208551 Japan; ^3^ Yamaha Motor Co., Ltd. 2500 Shingai, Iwata‐shi Shizuoka 4388501 Japan; ^4^ Department of Mechanical Engineering Faculty of Engineering Shizuoka University 3‐5‐1 Johoku, Naka‐ku, Hamamatsu‐shi Shizuoka 4328561 Japan

**Keywords:** diffusion, nitriding, shot particles transfer, surface modification, titanium alloys

## Abstract

Multifunctional surfaces are required to design safe engineering products for human lives. Heating in a nitrogen atmosphere (nitriding) improves the tribological properties but reduces the strength of titanium (Ti) alloys owing to grain coarsening. A rapid nitriding method for Ti alloys forms the nitrided layer on the surface of a Ti alloy by bombarding with commercially pure Ti fine particles with a nitrided phase at room temperature within a short period. Furthermore, fine grains of Ti alloy are formed in the nitrided layer because of the impact of the Ti particles. These results reveal that this room‐temperature method resolves the trade‐off between the rapid formation of a nitrided layer and the suppression of grain coarsening for Ti alloys.

## Graphical abstract

The mechanical and tribological properties are important for titanium alloys, which are used as biomaterials. Inspiration is taken from the transfer of fine particles to achieve rapid nitriding without heating. The developed room‐temperature methods result in the formation of a nitrided layer within a short period. The proposed method has the potential to improve various properties in other metallic materials.

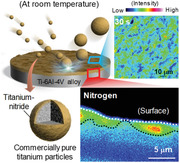

## 1 Formation of Nitrided Layer

**Figure 1:**
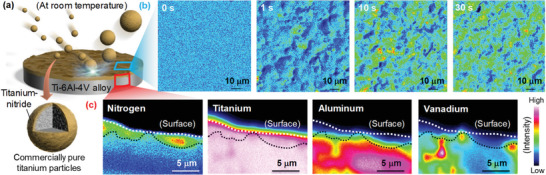
a) Schematic showing the rapid nitriding of a Ti–6Al–4V alloy specimen at room temperature by bombarding with CP Ti fine particles with a nitrided phase. b) N maps obtained by EPMA analysis of the surface of the modified Ti–6Al–4V. The N intensity detected by EPMA analysis of the modified surfaces tended to increase with increasing treatment time. c) Cross‐sectional elemental maps obtained by EPMA analysis of Ti–6Al–4V subjected to the surface treatment for 10 s at room temperature. A region showing high N and Ti intensities was observed near the modified surface, whereas the intensities of Al and V, which were components of the Ti–6Al–4V substrate, were low in the corresponding region. The formation of an inhomogeneous nitrided layer is attributed to the transfer of the CP Ti powder particles with a nitrided phase to the Ti–6Al–4V during particle bombardment.

Titanium (Ti) alloys are widely used in various products because of their high specific strength and good biocompatibility; however, Ti alloys exhibit inferior tribological properties compared with other materials. Nitriding, in particular, is an effective approach for improving tribological properties because it forms a titanium–nitride layer with high hardness, good thermal and chemical stability, and high wear resistance.^[^ ^1^ ^]^ In contrast, the strength of Ti alloys is reduced by grain coarsening during the nitriding process because nitriding of Ti alloys is generally performed at a high temperature (≈1173 K) to increase the nitrogen (N) diffusion rate.^[^ ^2^, ^3^, ^4^, ^5^, ^6^ ^]^ Thus, low‐temperature nitriding has been proposed as a method for suppressing the grain coarsening of Ti alloys and has already been found to increase the fatigue limit of Ti–6Al–4V alloy and form a hard surface layer.^[^ ^3^, ^4^ ^]^ Tang et al. reported that the Gibbs free energy change for the formation of nitrides became negative after a surface mechanical attrition pretreatment; they thereby successfully nitrided Fe at a lower temperature of 573 K.^[^ ^7^, ^8^ ^]^ However, reducing the nitriding temperature results in long nitriding durations because of the reduction of the N diffusion rate, which is problematic. Various rapid nitriding methods have therefore been developed.^[^ ^9^, ^10^, ^11^, ^12^ ^]^ For example, solar gas nitriding requires much less time (typically ≈15 min) than the conventional nitriding method; however, the maximum temperature is ≈1473 K.^[^ ^12^ ^]^ They still require that materials should be heated to extremely high temperatures even though the heating time is short. Therefore, in the present work, we developed a rapid nitriding method for Ti–6Al–4V alloy without heating. To form a nitrided layer on Ti alloy within a short period, we bombarded a Ti–6Al–4V substrate with commercially pure (CP) Ti fine particles with a nitrided phase at room temperature in air (**Figure** **1**a).^[^ ^13^ ^]^

Figure 1b shows N maps obtained by electron‐probe microscopic analysis (EPMA) of the surfaces of Ti–6Al–4V subjected to particle bombardment for various times at room temperature. The N intensity detected from the modified surfaces tended to increase with increasing treatment time. In contrast, the N intensity did not increase at the surface of Ti–6Al–4V subjected to the bombardment process using CP Ti particles with no nitrided phase for 30 s at room temperature (Figure S1, Supporting Information). Figure 1c shows cross‐sectional elemental maps for the Ti–6Al–4V specimen treated for 10 s at room temperature. A region with high N and Ti intensities was observed near the modified surface, whereas the intensities for aluminum (Al) and vanadium (V) elements, which were components of the Ti–6Al–4V substrate, were low in the corresponding region. The surface of the Ti–6Al–4V substrate bombarded with CP Ti particles with a nitrided phase was highly deformed in comparison with the surface of the substrate bombarded with CP Ti particles with no nitrided phase (Figure S2, Supporting Information). These phenomena indicate that the CP Ti particles with a nitrided phase were transferred to the Ti–6Al–4V substrate because of the impact of the particles. Thus, we found that the proposed process could form a nitrided layer on Ti alloys in air at room temperature.

## 2 Chemical State on Nitrided Layer

**Figure 2:**
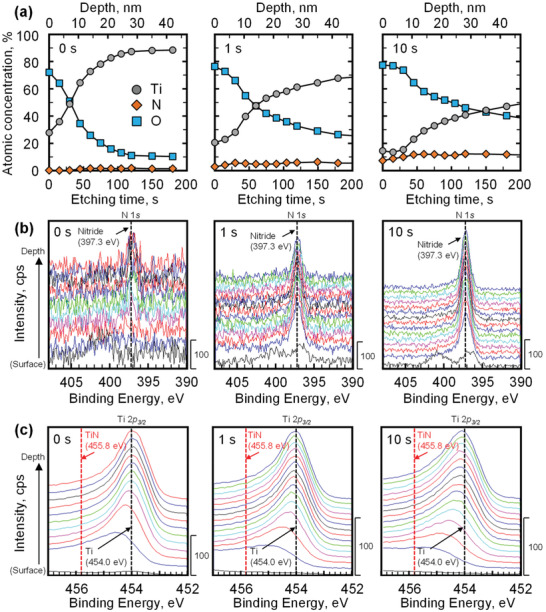
a–c) XPS analysis: a) depth profile, b) N 1*s* spectra, and c) Ti 2*p*_3/2_ spectra of the surfaces of untreated and modified Ti–6Al–4V treated for 1 and 10 s at room temperature. The N intensities in the spectra of the modified Ti–6Al–4V were greater than those in the spectra of the untreated sample. The N concentration of the modified Ti–6Al–4V tended to increase with increasing treatment time. Peaks due to TiN were not detected from the treated samples. These results are attributed to the transfer of the CP Ti particles with a nitrided phase to the Ti–6Al–4V substrate during the bombardment process.

To examine the N chemical states on the surface‐modified layer, the processed Ti–6Al–4V surfaces were analyzed by X‐ray photoelectron spectroscopy (XPS). **Figure** **2**a shows the relationship between the atomic concentration and depth from the surface for each Ti–6Al–4V specimen. The N concentrations for the modified Ti–6Al–4V samples were higher than that for the untreated sample and tended to increase with increasing treatment time. Figure 2b,c shows the XPS analysis results for N and Ti on the modified surfaces, where the vertical axis is related to the depth from the modified surface. Peaks for N 1*s* were detected in the spectra of the modified surfaces, whereas peaks for TiN were not detected. These results are attributed to the transfer of CP Ti particles with a nitrided phase to the Ti–6Al–4V substrate during the bombardment.

## 3 Effect of Treatment Time on Thickness of Nitrided Layer

**Figure 3:**
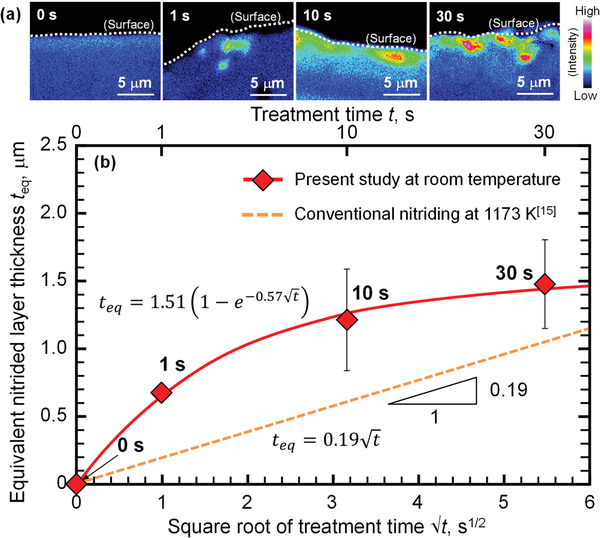
a) N maps obtained by EPMA analysis of the cross section of modified Ti–6Al–4V treated for 0, 1, 10, and 30 s. b) Relationship between the equivalent thickness, *t*_eq_, of the nitrided layer formed on the surface of modified Ti–6Al–4V and the square root of the treatment time, √*t*. The N intensities of the modified Ti–6Al–4V were greater than those of the untreated sample. The N concentration of the modified Ti–6Al–4V tended to increase with increasing treatment time. The nitride layer formed by the developed method was thicker than that of the layer formed by conventional nitriding at 1173 K at a comparable treatment time.^[^ ^15^ ^]^ The formation rate of the nitrided layer, d*t*_eq_/d√*t*, in conventional nitriding at 1173 K had a constant value of 0.19 µm s^−1/2^,^[^ ^15^ ^]^ whereas d*t*_eq_/d√*t* in the developed method depended on the treatment time. The developed process can achieve rapid nitriding of a Ti alloy within a short period at room temperature because of the transfer and mechanical alloying of CP Ti particles with a nitrided phase.

To quantitatively investigate the effect of the treatment time on the thickness of the inhomogeneous nitrided layer formed on Ti–6Al–4V, we used EPMA to analyze the N intensity on a cross section of the modified Ti–6Al–4V specimens treated for 0, 1, 10, and 30 s (**Figure** **3**a). Figure 3a demonstrates that the equivalent thickness of the nitrided layer could be calculated from Equation (1) under the assumption that the nitrided layer was uniformly formed on the surface of the Ti alloy:^[^ ^14^ ^]^

(1) $$\begin{array}{c} t_{\text{eq}}={\text{area}}_{N}/b \end{array}$$

where *t*_eq_ is the equivalent thickness of the nitrided layer (µm), area_N_ is the area in which the N intensity was higher than in the untreated sample (µm^2^), and *b* is the width of the specimen in the analyzed area. Figure 3b shows the relationship between the *t*_eq_ of the nitrided layer formed on the surface‐modified Ti–6Al–4V and the square root of the treatment time, √*t*. The nitrided layer thickness tended to increase with increasing treatment time in both series. The nitrided layer on the surface treated using the developed room‐temperature process was thicker than that formed by conventional nitriding at 1173 K at a comparable treatment time (Figure 3b).^[^ ^15^ ^]^

The nitrided layer thickness after conventional nitriding at 1173 K has been reported to be proportional to the square root of the treatment time,^[^ ^15^ ^]^ whereas the thickness of the nitrided layer formed by the method developed in the present work increased nonlinearly. To elucidate the formation mechanism of the nitrided layer, the equations showing the relationship between the thickness of the nitrided layer and the treatment time in both series are shown in Figure 3b. The formation rate for the nitrided layer, d*t*_eq_/d√*t*, in conventional nitriding at 1173 K has been reported to exhibit a constant value of 0.19 µm s^−1/2^,^[^ ^15^ ^]^ whereas the d*t*_eq_/d√*t* (=0.86*e* ^−0.57√^ *^t^*) in the developed method varies with the treatment time. This result means that the nitrided layer in conventional nitriding forms only through N diffusion, whereas that in the developed method forms mainly through the transfer of shot particles.^[^ ^16^ ^]^ Furthermore, the d*t*_eq_/d√*t* at *t* = 0 (0.86 µm s^−1/2^)(i.e., at the early stage) was 4.5 times greater than that in conventional nitriding at 1173 K. Figure 3b also reveals that d*t*_eq_/d√*t* (=0.86*e* ^−0.57√^ *^t^*) tended to decrease with increasing treatment time. The nitrided particles collided with the Ti alloy; as bombardment continued, the particles collided with a nitrided surface with increasing hardness, which resulted in a reduction of the formation rate because of the increase in deformation resistance with increasing treatment time. Thus, the developed process achieved rapid nitriding of the Ti alloy within a short period at room temperature because of the transfer and mechanical alloying of the CP Ti particles with a nitrided phase.

To verify this assumption, we conducted more detailed analyses and hardness tests on the surface of the modified Ti–6Al–4V. Figure S3, Supporting Information, shows a scanning electron microscopy (SEM) image corresponding to the N map in Figure 3a and a more detailed SEM image of the cross section of the modified Ti–6Al–4V specimen. These figures demonstrate that the N intensity detected by EPMA analysis was high near the CP Ti particles with a nitrided phase transferred to the Ti–6Al–4V substrate. Furthermore, the hardness value measured at a surface dent formed by the bombardment of CP Ti particles with a nitrided phase was much higher than those for the untreated and the sample bombarded with CP Ti particles with no nitrided phase (Figure S4, Supporting Information). These results indicate that the modified Ti–6Al–4V was mechanically alloyed because of the impact of the CP Ti particles with a nitrided phase. Consequently, both mechanical alloying and the transfer of CP Ti particles were found to have occurred during the bombardment process at room temperature.

## 4 Microstructural Characterization

**Figure 4:**
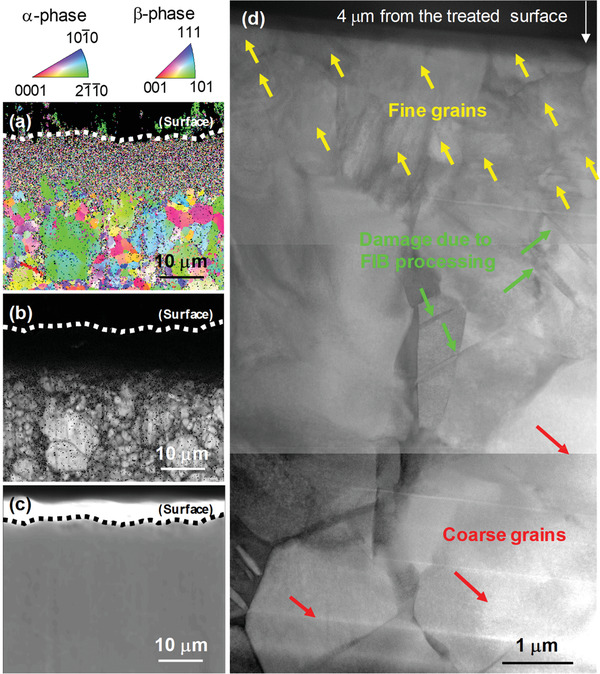
a) IPF map, b) IQ map, c) SEM image, and d) cross‐sectional STEM image, as obtained by EBSD and STEM analyses, of Ti–6Al–4V subjected to surface modification for 30 s. A fine‐grained structure was formed near the treated surface of Ti–6Al–4V, as represented by yellow arrows. The developed surface modification could not only form a nitrided layer but also refine the microstructure of the Ti alloy at room temperature. The developed method resolves the trade‐off between the rapid formation of a nitrided layer and the suppression of grain coarsening for Ti alloys.

We characterized the microstructures of the Ti alloy subjected to the rapid nitriding treatment using electron backscatter diffraction (EBSD) because Ti alloys subjected to the conventional nitriding process tend to exhibit grain coarsening. **Figure** **4** shows an inverse pole figure (IPF) map and image quality (IQ) map of Ti–6Al–4V subjected to surface modification for 30 s. Each map in Figure 4a–c corresponds to the same region. No clear IPF or IQ maps were observed from the outermost surface at a depth of ≈8 µm (Figure 4a,b). In the SEM image, the outermost edge part was observed (Figure 4c); therefore, a highly strained region would be formed near the Ti–6Al–4V surface modified by the collision of the CP Ti particles with a nitrided phase. To analyze the microstructure near the surface in greater detail, we characterized the corresponding region by preparing a specimen using SEM‐focused ion beam (FIB) and observing it using scanning transmission electron microscopy (STEM). Although the specimen for STEM observation was carefully prepared using the FIB method, slight damage due to the FIB processing was observed, as represented by the green arrows (Figure 4d). On the side less affected by the surface modification, the crystal grains (represented by red arrows) were coarse; by contrast, fine grains (represented by the yellow arrows) were observed on the opposite side. Thus, we speculate that the microstructure near the treated surface was refined through dynamic recrystallization or grain subdivision.^[^ ^17^, ^18^ ^]^

The effect of the developed room‐temperature process on the basic properties of the Ti–6Al–4V was examined. The 0.2% proof stress for the modified Ti–6Al–4V, as obtained via four‐point bending tests, was higher than those for the untreated and the conventional nitrided Ti–6Al–4V, whose strength was compromising (Figure S5, Supporting Information). Furthermore, the compressive residual stress in the sample processed by CP Ti particles with a nitrided phase was higher than those in the untreated and the sample bombarded with CP Ti particles with no nitrided phase (Figure S6a, Supporting Information). In contrast, tensile residual stress, which reduces the fatigue strength of materials, was generated on the conventional nitrided surface. The full‐width at half‐maximum (FWHM) value for the sample bombarded with CP Ti particles with a nitrided phase was also higher than that for other samples because of the formation of fine grains (Figure S6b, Supporting Information). These results indicate that the fatigue strength of the modified Ti–6Al–4V would be higher than that of the conventional nitrided sample because of the suppression of fatigue crack initiation and propagation. Furthermore, the friction coefficient for the modified Ti alloy was lower than that for the untreated sample in the early stage during ball‐on‐disk dry friction tests (Figure S7, Supporting Information). Thus, the developed room‐temperature process improved the various properties of the Ti alloys by rapidly forming of a nitrided layer.

## 5 Conclusion

We have demonstrated that the developed rapid nitriding method can form a nitrided layer on the surface of Ti–6Al–4V within a short period at room temperature and under atmospheric pressure because of mechanical alloying and the transfer of the CP Ti particles with a nitrided phase. More importantly, in the early stage, the formation rate of the developed room‐temperature process is higher than that of the conventional nitriding method conducted at 1173 K, even though the Ti alloy is not heated. Furthermore, in general, conventional nitriding causes grain coarsening because of the high temperatures, whereas the developed method can form a fine‐grained structure on the Ti alloy without heating. The developed method solves the trade‐off between rapid formation of a nitrided layer and suppression of grain coarsening in Ti alloys.

## 6 Experimental Section

### Materials

The Ti alloy used in the present work was Ti–6Al–4V (ELI grade) with a chemical composition of 6.31% Al, 4.13% V, 0.12% Fe, 0.002% H, 0.006% N, 0.11% O, and 0.024% C by mass, with Ti comprising the balance. This material contained both the equiaxed α‐phase and the β‐phase.^[^ ^4^, ^14^ ^]^ Ti–6Al–4V ELI plates with a thickness of 11 mm were machined into 1.5 mm‐thick sheets and then cut into 3 × 20 mm^2^ specimens using a wire electrical discharge machine. After machining, the specimens were mirror‐polished to a thickness of 1 mm with emery paper (from #120 to #4000) using a SiO_2_ suspension. The Ti plate specimens had a Vickers hardness of 355.2 ± 4.0 HV, as measured for a polished surface using an indentation force of 0.098 N and a load holding time of 10 s (*n* = 30).

### Methods

CP Ti particles (25.6 µm particle diameter, ranging from 0 to 45 µm; TILOP‐45 fabricated in Osaka Titanium Technologies Co., Ltd.) with a nitrided phase^[^ ^13^ ^]^ was used in the present work. Figure S8, Supporting Information, shows a cross‐sectional SEM image of a CP Ti particle with a nitrided phase. The particles evidently had a smooth spherical shape. As shown in Figure S9, Supporting Information, a high‐N‐intensity region was observed near the surface of the CP Ti particle with a nitrided phase. Figure S10, Supporting Information, shows the depth profiles for N elements analyzed by EPMA in the cross section of CP Ti particle with a nitrided phase. The N intensity at the vertical axis in the present study corresponded to the N concentration. The N concentration in CP Ti particles varied as a function of position, and a high‐N‐intensity region was observed near the surface of the CP Ti particle with a nitrided phase in comparison with the N intensity detected inside the material. The polished Ti–6Al–4V specimens were subjected to particle bombardment for 1, 10, or 30 s by CP Ti particles with a nitrided phase at room temperature in air using a direct‐pressure‐type apparatus. Samples bombarded with CP Ti particles with no nitrided phase and treated with conventional nitriding at 1123 K for 18 000 s were also prepared for comparison. The bombarding pressure was 0.5 MPa, and the nozzle distance was 30 mm. The N mapping images were obtained for the surfaces of the treated Ti–6Al–4V specimens over a 40 000 µm^2^ region using EPMA at an acceleration voltage of 15 kV. In addition, cross‐sectional elemental mapping images were obtained by EPMA analysis for Ti–6Al–4V specimens treated for various times at room temperature over a 240 µm^2^ region.

To examine the N chemical states on the surface‐modified layer, the processed Ti–6Al–4V surfaces were analyzed by XPS (AXIS‐ULTRA, Shimadzu/KRATOS) using Al Kα radiation (1486.6 eV). The energy resolution in the XPS measurements, which was measured on the basis of the FWHM of the Ag 3*d*_5/2_ peak, was 0.48 eV. The anode high‐tension and emission current were 15 kV and 10 mA, respectively. Depth profiles were obtained by Ar sputtering at an etching rate of 14 nm min^−1^. The source high‐tension and extractor current were 5 kV and 150 µA, respectively. Furthermore, the crystal structure of the treated Ti alloys was examined by EBSD at an accelerating voltage of 15 kV on a cross section of the treated Ti.

The area_N_ was calculated from N maps obtained by EPMA analysis of the cross section of modified Ti–6Al–4V alloy. The equivalent thickness of the nitrided layer was calculated from Equation (1), where *t*_eq_ was the equivalent thickness of the nitrided layer (µm), and *b* was the width of the specimen in the analyzed area (18.0 µm in the present study). The area_N_ was determined from the N map, as shown in Figure S11, Supporting Information. Figure S11a, Supporting Information, shows an original cross‐sectional N map of the specimen treated for 10 s, whereas Figure S11b, Supporting Information, shows a modified N map that was reduced on the basis of the N intensity in the map of the untreated sample. The area in which the N intensity was higher than in other areas was measured as area_N_, as represented by the white arrows in the modified N map.

To analyze the characteristic structure of the modified Ti alloys near the surface in greater detail, the corresponding region was processed using a SEM‐FIB method and observed using STEM (JEM‐2100F, JEOL) operated at 200 kV and equipped with a Cs‐corrector (spherical aberration corrector).

The 0.2% proof stress of the Ti alloys was measured using an electrodynamic apparatus under four‐point bending in air at room temperature. A four‐point bending moment was applied to the specimens, where the distance between the inner and outer pins was 5.0 and 15.0 mm, respectively. Tensile stress and the compressive stress always appeared simultaneously on either side of the specimen under bending. In the present study, strain at tensile side was measured using strain gauges.

The residual stress and FWHM values were measured using an X‐ray stress analyzer (µ‐X360s, Pulstec Industrial Co., Ltd.) via the peak‐top method in the longitudinal direction after measurement of the (103) strain between lattice planes with the V Kα line generated at 30 kV and 1.2 mA. A region of ⌀2 mm was measured.

Ball‐on‐disk dry friction tests were performed at ambient temperature using 3 mm steel ball bearings to examine the effects of the developed room‐temperature process on the tribological properties of the Ti alloys. The nominal applied force was 10 N, the sliding distance was 8 m, and the movement rate was 12 mm s^−1^.

## Conflict of Interest

The authors declare no conflict of interest.

## Supporting information

Supporting Information

## Data Availability

Research data are not shared.
