# Supplementary material for: Rapid Nitriding of Titanium Alloy with Fine Grains at Room Temperature
Source: Adv Mater. 2021 May 3;33(20):2008298. doi: 10.1002/adma.202008298 (PMC11468969; doi:10.1002/adma.202008298)
Supplement: Supplementary file 1 — Supporting Information [file ADMA-33-2008298-s001.pdf]

© 2021 Wiley-VCH GmbH

ADVANCED MATERIALS

Supporting Information

for *Adv. Mater.,* DOI: 10.1002/adma.202008298

Rapid Nitriding of Titanium Alloy with Fine Grains at Room Temperature

*Keisuke Fujita, Masataka Ijiri, Yoichi Inoue, and Shoichi Kikuchi**

Supporting Information

Rapid Nitriding of Titanium Alloy with Fine Grains at Room Temperature

*Keisuke Fujita, Masataka Ijiri, Yoichi Inoue, Shoichi Kikuchi**

Figure 3 shows the equations showing the relationship between the thickness of the nitrided layer and the treatment time in both series are shown as follows:

Conventional nitriding at 1173 K: $t_{eq} = 0.19\sqrt{t}$ (S1)

Developed room-temperature method: $t_{eq} = 1.51(1 - e^{-0.57\sqrt{t}})$ (S2)

Equation S2 indicates that the thickness of the nitrided layer then approached 1.51 μm (t_{eq}) at $t = \infty$.

The method for producing the specimen for STEM observation is generally performed by electrochemical polishing; however, it has previously reported that electrochemical polishing method causes the change in the crystal structure or the formation of intermetallic compounds due to the hydrogen contained in the sample during the spontaneous transformation and polishing of the thin film. Therefore, the specimen for STEM observation was prepared using a FIB. The entire image of the material shows a slightly processed layer by FIB; however, the disturbance due to sample preparation seems to be lesser than that by electrochemical polishing.[1]-[5]

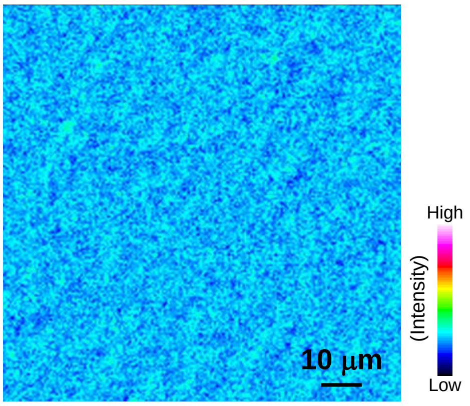

Figure S1. N map obtained by EPMA analysis of the surface treated with CP Ti particles with no nitrided phase for 30 s.

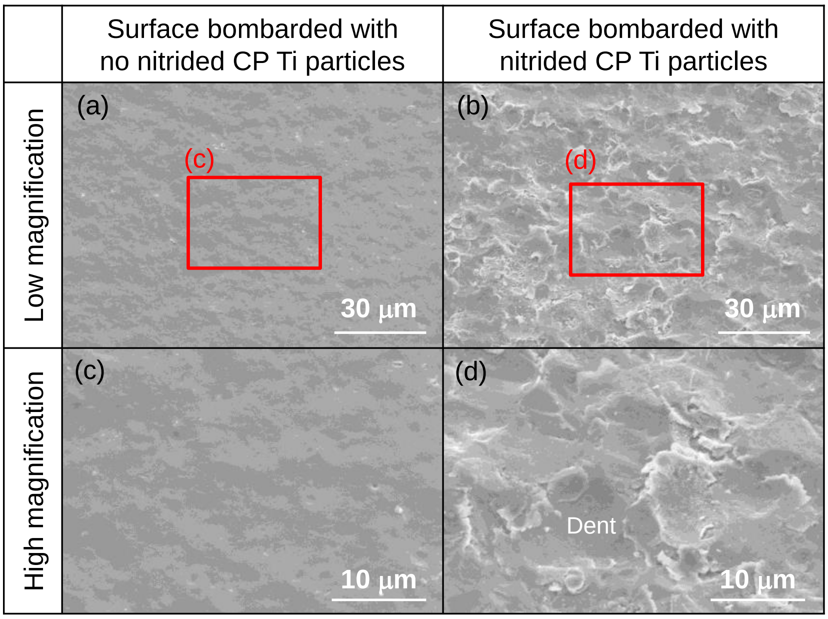

Figure S2. SEM micrographs of the surface treated with CP Ti particles with no nitrided phase and with a nitrided phase for 30 s.

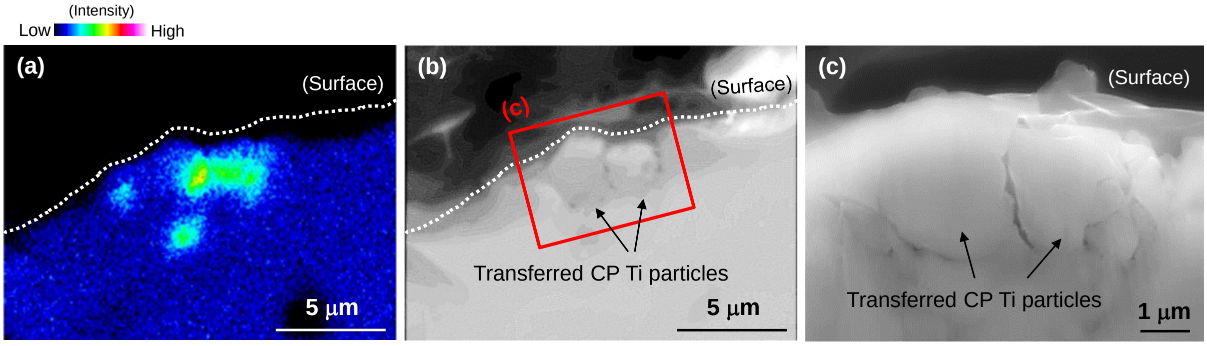

Figure S3. Cross-sectional (a) N map, (b) SEM micrograph and (c) SEM micrograph observed at higher magnification for the Ti-6Al-4V specimen treated with CP Ti particles with a nitrided phase for 1 s.

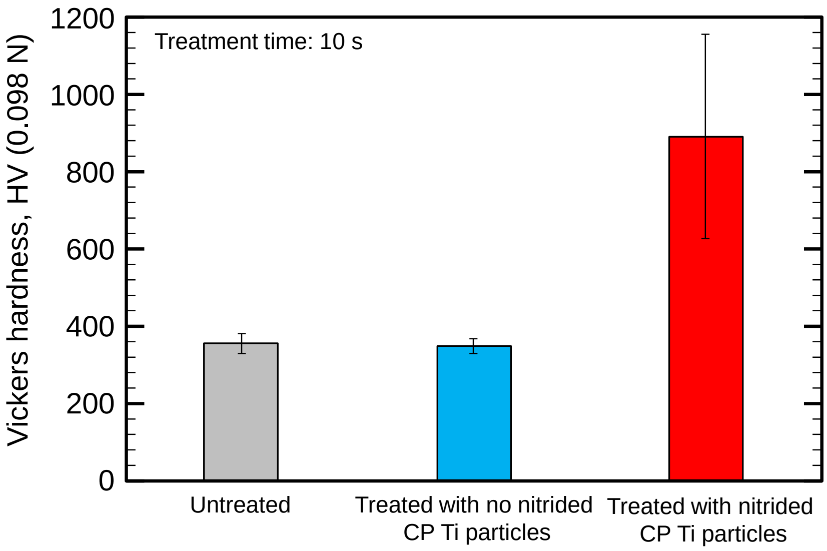

Figure S4. Vickers hardness for the Ti-6Al-4V specimens measured at the top surface.

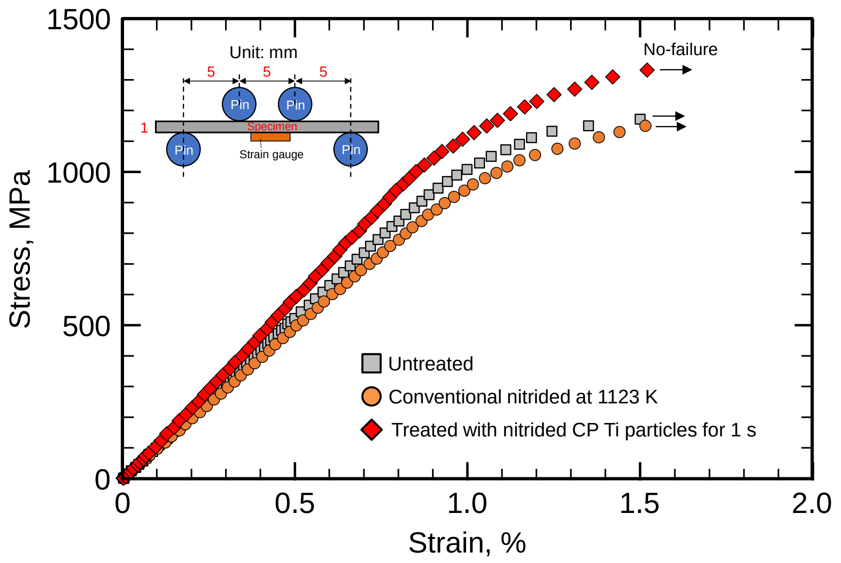

Figure S5. Stress-strain curves for the Ti-6Al-4V specimens under four-point bending.

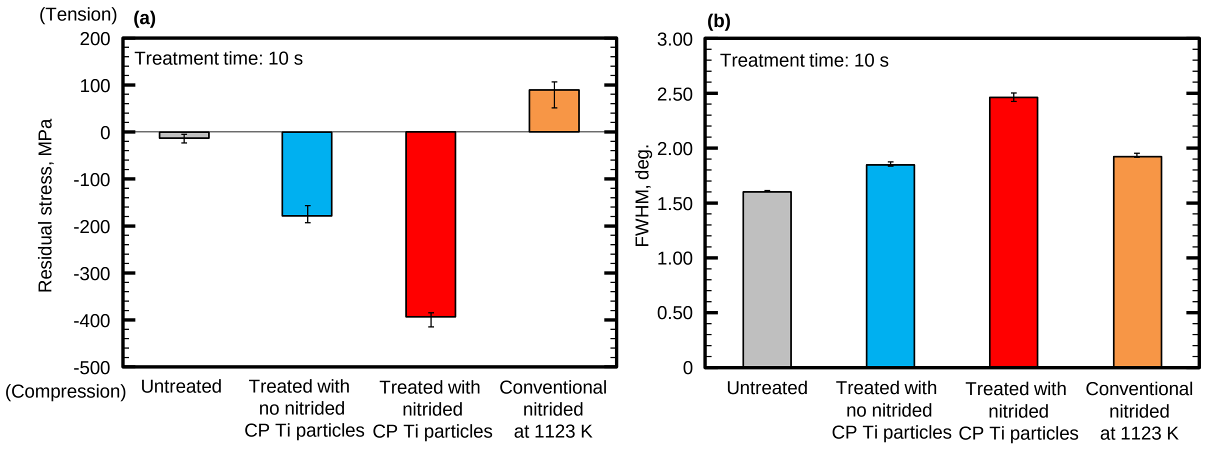

Figure S6. Results of measuring (a) residual stress and (b) FWHM values for the Ti-6Al-4V specimens at the top surface.

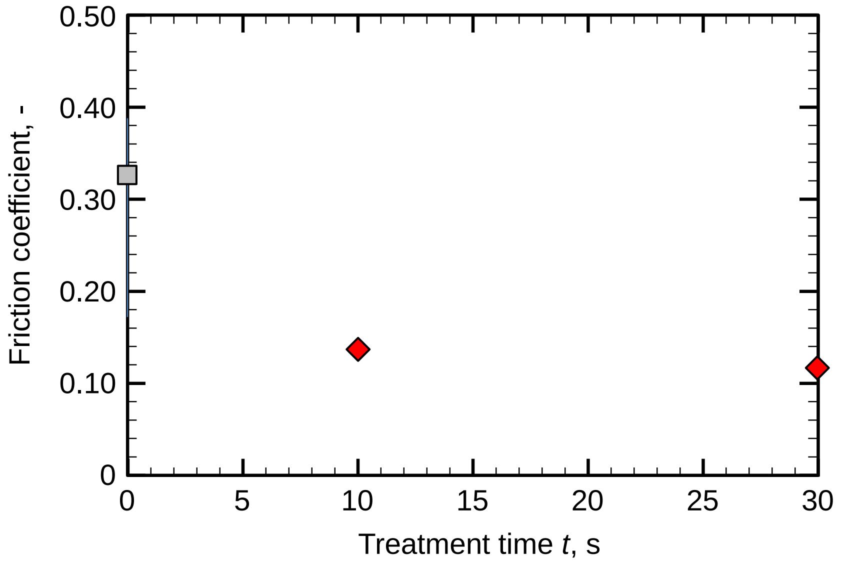

Figure S7. Friction coefficient for the Ti-6Al-4V specimens treated with CP Ti particles with a nitrided phase in the early stage.

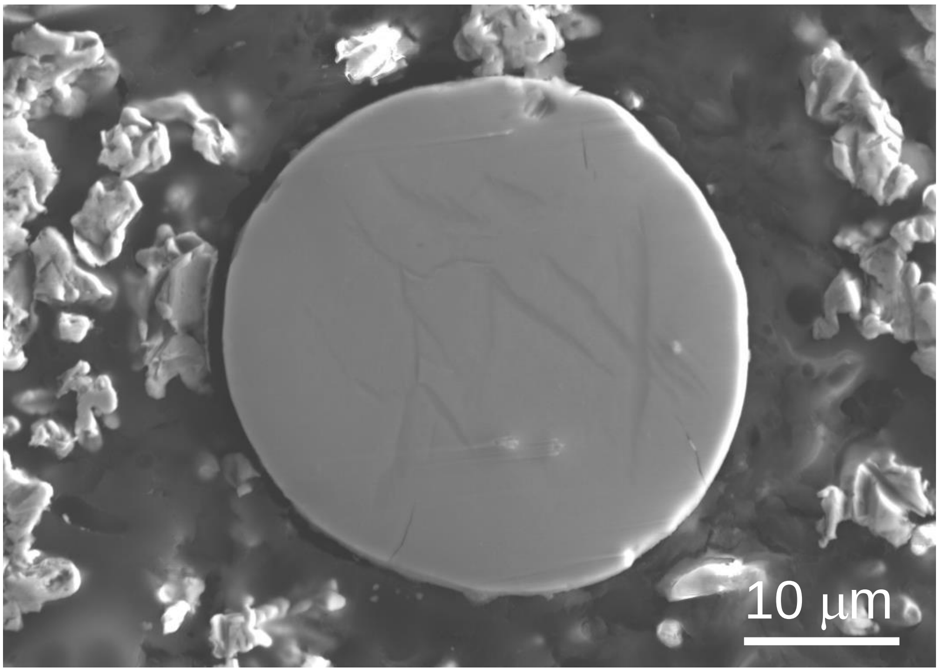

Figure S8. Cross-sectional SEM micrograph of a CP Ti particle with a nitrided phase.

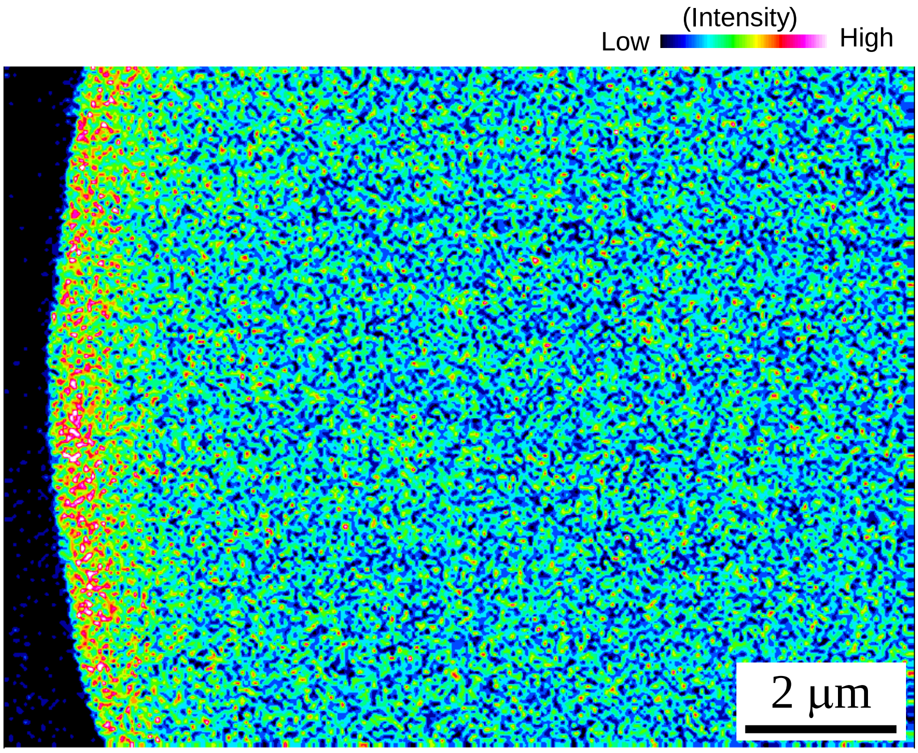

Figure S9. Cross-sectional N map of a CP Ti particle with a nitrided phase.

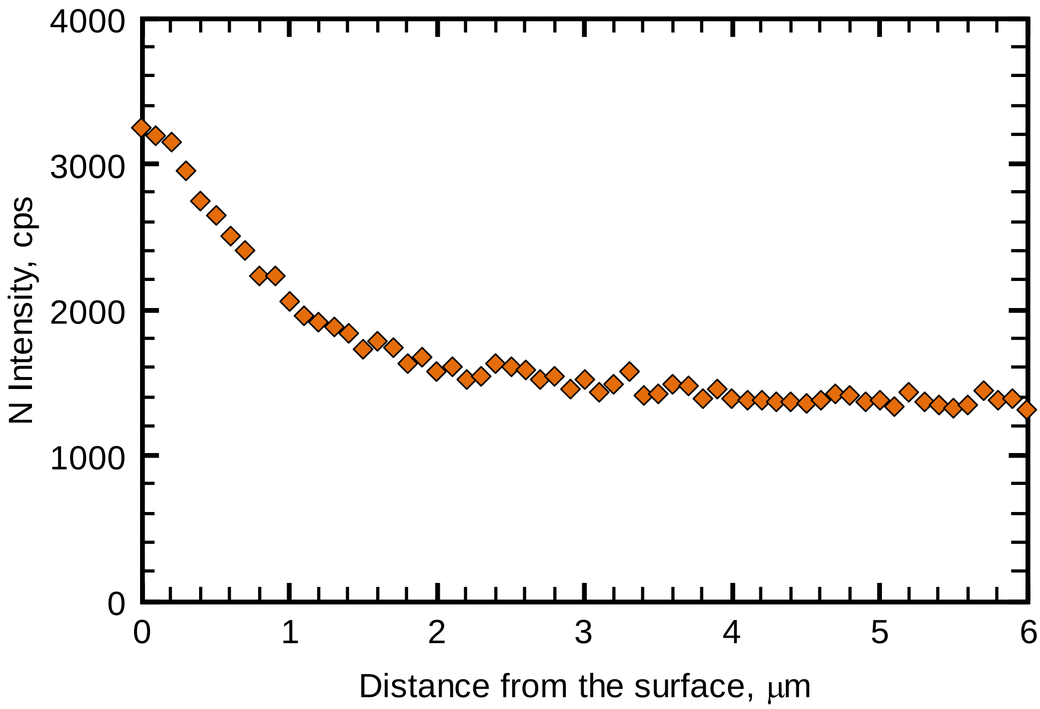

Figure S10. Depth profile for N elements analyzed by EPMA in the cross section of CP Ti particle with a nitrided phase.

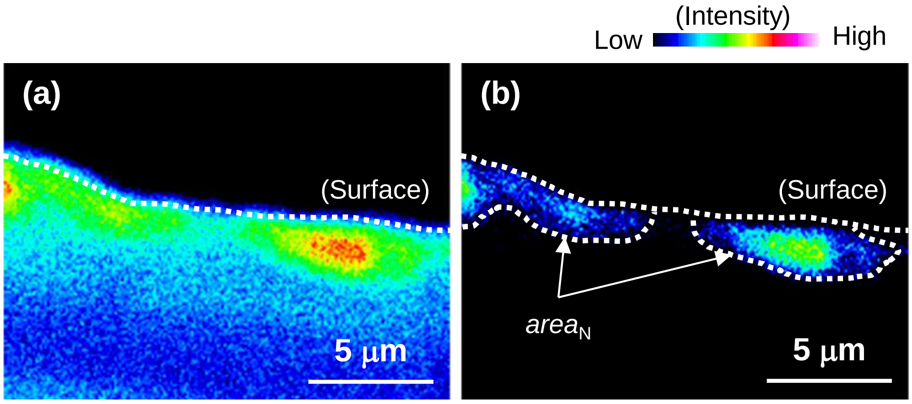

Figure S11. a) Original and b) modified N maps, which was reduced on the basis of the N intensity in the map of the untreated sample, obtained by EPMA analysis of the cross section of modified Ti-6Al-4V treated for 10 s.

References

[1] M. J. Blackburn and J. C. Williams, *Trans. Met. Soc. AIME* **1967**, *239*, 287.

[2] M. J. Blackburn and J. C. Williams, *Trans. Met. Soc. AIME* **1968**, *242*, 2461.

[3] R. A. Spurling, C. G. Rhodes and J. C. Williams, *Met. Trans.* **1974**, *5*, 2597.

[4] R. A. Spurling, *Met. Trans. A* **1975**, *6A*, 1660.

[5] M. Ijiri, Y. Tomita, T. Ishikawa and Y. Takemoto, *J. Jpn. Inst. Met. Mater.* **2016**, *80*, 547.
